# Mitral valve replacement in infants and younger children

**DOI:** 10.1038/s41598-021-94779-0

**Published:** 2021-07-27

**Authors:** Ahmed F. Elmahrouk, Mohamed H. Mashali, Mohamed F. Ismail, Amr A. Arafat, Rawan M. Alamri, Haysam A. Baho, Mohammad S. Shihata, Ahmed A. Jamjoom

**Affiliations:** 1 Division of Cardiac Surgery, Cardiovascular Department, King Faisal Specialist Hospital and Research Center, MBC J-16, P.O. Box: 40047, Jeddah, 21499 Saudi Arabia; 2 Cardiothoracic Surgery Department, Tanta University, Tanta, Egypt; 3 Pediatric Cardiology Department, King Faisal Specialist Hospital and Research Center, Jeddah, Saudi Arabia; 4 Department of Pediatrics, Faculty of Medicine, Cairo University, Cairo, Egypt; 5 Cardiothoracic Surgery Department, Mansoura University, Mansoura, Egypt

**Keywords:** Cardiac device therapy, Cardiovascular biology, Paediatric research

## Abstract

Data on mitral valve replacement (MVR) in young children is still limited. Our objective was to evaluate MVR in children below 5 years and identify factors affecting the outcomes. This retrospective study included 29 patients who had MVR from 2002 to 2020. We grouped the patients into two groups according to their age: age ≤ 24 months (n = 18) and > 24 months (n = 11). Primary cardiac diagnoses were Shone complex (n = 7; 24%), isolated congenital mitral valve abnormality (n = 11; 38%), and complete atrioventricular septal defect (n = 3; 10%). The median age was 19 month (25th–75th percentile: 11–32) and 59% were females (n = 17). The hemodynamic lesions were mitral regurgitation in 66%, mitral stenosis in 10%, and combined mitral stenosis and regurgitation in 24% of the patients. St. Jude mitral valve was the most common valve implanted (n = 19, 66%), followed by CarboMedics in 21% of the patients (n = 6). The mitral valve was implanted in the supra-annular position in 6 cases (21%). Preoperative and operative data were comparable between both groups. There was no association between valve size and position with postoperative heart block (P > 0.99, for both). The median follow-up duration was 19.4 months (8.6–102.5). Nine patients had mitral valve reoperation, six had MVR, and three had clot removal from the mitral valve. There was no effect for age group on reoperation (SHR 0.89 (95% CI 0.27–2.87), P = 0.84). Valve size significantly affected reoperation (SHR 0.39 (95% CI 0.18–0.87), P = 0.02). The supra-annular position was associated with an increased risk of reoperation (SHR 3.1 (95% CI 1.003–9.4), P = 0.049). There was no difference in survival according to the age (Log-rank P = 0.57) or valve size (Log-rank P = 0.66). Mitral valve replacement in children is associated with low morbidity and mortality. The risk of reoperation could be affected by the valve size and position rather than the age.

## Introduction

Mitral valve repair is the recommended treatment strategy for infants and children with surgical mitral valve (MV) disease; however, mitral valve replacement (MVR) is performed when mitral valve repair is not anatomically feasible^1^ The decision to perform MVR in young children is difficult^2^. Studies reporting the outcomes of MVR in children are limited by the sample size, inclusion of a wide range of ages, and the single-center experience^3^.

MVR in children has several limitations, and it is associated with a high mortality rate ranging from 10 to 36%^4^. Moreover, long-term anticoagulation therapy is associated with increased morbidity, in addition to the risk of repeat MVR as the child grows^5^. The outcomes after MVR in children under 5 years are still controversial. We aimed to evaluate our experience with mitral valve replacement in infants and young children less than 5 years old and identify factors affecting the outcomes.

## Patient and methods

### Design and patients

We performed a retrospective study including patients who underwent MVR at King Faisal Specialist and Research Center, Jeddah, between May 2002 and December 2020. We included patients aged 5 years or younger. Patients who had prior MVR performed outside the center were excluded. All patients had elective MVR. We grouped the patients into two groups according to their age: age ≤ 24 months (n = 18) and age > 24 months and ≤ 60 (n = 11). Primary cardiac diagnoses were Shone complex in seven cases (24%), isolated congenital MV abnormality in eleven cases (38%), and complete atrioventricular septal defect in three cases (10%). The study was approved by the Institutional Review Board of King Faisal Specialist Hospital and Research Center with (Registration number: 2020-105). All procedures in this study were carried out after prior informed consent from patients' guardians, in accordance with relevant guidelines and regulations. The need for the guardians' consent for publication was waived due to the retrospective nature of the study.

### Study data and outcomes

We collected the preoperative, operative, and postoperative data required for this study from electronic and paper medical records. Operative data included the mitral valve position, type, and size. Study outcomes were hospital complications, reoperation, and long-term survival. Hospital outcomes were defined as those occurring during the indexed hospitalization or within 30 days from surgery. Follow-up of the patients was recorded until December 2020.

### Postoperative anticoagulation regimen

Postoperatively, patients were anticoagulated with warfarin with a target international normalization ratio (INR) of 2.5–3.5. We started heparin infusion within 24 h postoperatively as bridging anticoagulation. INR was measured and reported daily until the target level was achieved, then every seven days following discharge. Patients were followed after discharge in the anticoagulation outpatient clinic. Two patients were shifted to low molecular weight heparin due to postoperative bleeding with Activated Factor X monitoring and shifted back to warfarin after completing the first year of life. Supplementary antiplatelet therapy "Aspirin" was used in the first six months postoperatively in selected cases.

### Statistical analysis

We compared qualitative data using the Chi-square or Fisher exact test if the expected frequency was less than 5, and data were presented as numbers (%). The quantitative variables were compared using the *t* test if normally distributed or the Mann–Whitney test if non-normally distributed. Normally distributed data were presented as mean and standard deviation and non-normal data as median (25th–75th percentiles). Correlation was assessed with Spearman test. Time to event outcomes were compared using the Log-rank test, and their distribution was plotted with the Kaplan–Meier method. Death was considered a competing risk for mitral valve reoperations. The Fine and Gray method was used to perform competing risk regression, and the cumulative incidence was plotted. We used STATA 16.1 (Stata Corp, College Station, TX, USA) to perform all analyses.

### Ethics approval

The study was approved by the institutional review board (IRB) of King Faisal specialist hospital and research center, Jeddah, Saudi Arabia, under approval number: IRB# 2020-105.

### Consent to participate

All procedures in this study were carried out after prior informed consent from patients' guardians, in accordance with relevant guidelines and regulations.

## Results

### Preoperative and operative data

The median age of our sample was 19 month (25th–75th percentile: 11–32); 59% were females (n = 17). Mitral valve pathology included parachute MV in 4 cases (14%), absent and short chordae in 3 cases (10%). Eight patients (28%) had previous operative attempts of mitral valve repair. Mitral valve regurgitation was the most common lesion. St. Jude mitral valve was the most common valve implanted (n = 19, 65.5%), followed by CarboMedics in 21% of the patients (n = 6). The size of MV used was less than 17 mm in 38%, 17–21 mm in 45%, and more than 21 mm in 17% of cases. The mitral valve was put in supra-annular position in six cases (21%). There was no correlation between the valve size, age (P = 0.80) or body surface area (P = 0.09). Preoperative and operative data are presented in Table 1.

**Table 1:** Preoperative and operative data.

	All patients (n = 29)	Age ≤ 24 months (n = 18)	Age > 24 months and ≤ 60 (n = 11)	P
**Preoperative data**				
Age (months)	19 (11–32)	11 (6–15)	40 (30–48)	< 0.001
Female	17 (58.6%)	9 (50%)	8 (72.7%)	0.27
Body weight (Kg)	9.3 (8.3–13)	9 (7.9–11)	12.2 (10.8–13.3)	0.08
Body surface area (m^2^)	0.43 (0.30–0.53)	0.38 (0.27–0.46)	0.52 (0.44–0.59)	0.03
Associated non-cardiac anomalies	3 (10.3%)	1 (5.6%)	2 (18.2%)	0.54
Non cardiac surgery	3 (10.3%)	3 (16.7%)	0	0.27
		Orchidopexy		
		Pyloroplasty		
		Ilium perforation		
Down syndrome	4 (13.8%)	3 (16.7%)	1 (9.1%)	> 0.99
Diagnosis				0.75
Shone's complex	7 (24.1%)	3 (16.7%)	4 (36.4%)	
Congenital MR	11 (37.9%)	8 (44.4%)	3 (27.3%)	
AV canal	3 (10.3%)	2 (11.1%)	1 (9.1%)	
Others*	8 (27.6%)	5 (27.8%)	3 (27.3%)	
**MV pathology**				
Parachute MV	4 (13.8%)	3 (16.7%)	1 (9.1%)	> 0.99
Displaced papillary muscle	1 (3.5%)	0	1 (9.1%)	0.38
Absent or short chordae	3 (10.3%)	2 (11.1%)	1 (9.1%)	> 0.99
MV lesion				0.71
Stenosis	3 (10.3%)	1 (5.6%)	2 (18.2%)	
Regurgitation	19 (65.5%)	12 (66.7%)	7 (63.6%)	
Double	7 (24.1%)	5 (27.8%)	2 (18.2%)	
PHT	4 (13.8%)	4 (22.2%)	0	0.27
Mean gradient	16 (7–20)	7 (6–13)	19.5 (18–22)	0.002
**Operative details**				
Posterior leaflet preservation	3 (10.3%)	2 (11.1%)	1 (9.1%)	> 0.99
Size of the valve (mm)				
16	5 (17.24%)	3 (16.67%)	2 (18.18%)	0.66
17	6 (20.69%)	4 (22.22%)	2 (18.18%)	
18	2 (6.90%)	1 (5.56%)	1 (9.99%)	
19	5 (17.24%)	4 (22.22%)	1 (9.09%)	
21	6 (20.69%)	2 (11.11%)	4 (36.36%)	
23	2 (6.90%)	2 (11.11%)	0	
24	1 (3.45%)	1 (5.56%)	0	
25	1 (3.45%)	1 (5.56%)	0	
27	1 (3.45%)	0	1 (9.09%)	
CPB (min)	112.5 (94–160)	118 (79–178)	106 (94–130)	0.72
Cross-clamp (min)	78 (62–98)	78 (54–120)	78 (71–94)	0.97
Type of the valve				0.88
St. Jude	19 (65.5%)	12 (66.7%)	7 (63.6%)	
Carbomedics	6 (20.7%)	4 (22.2%)	2 (18.2%)	
St. Jude aorta	2 (6.9%)	1 (5.6%)	1 (9.1%)	
On-X	1 (3.5%)	1 (5.6%)	0	
Tissue	1 (3.5%)	0	1 (9.1%)	
Supra-annular position	6 (20.7%)	5 (27.8%)	1 (9.1%)	0.36

Continuous data were presented as median (25th–75th percentiles) and categorical data as number (%).

*AV* atrioventricular, *CPB* cardiopulmonary bypass, *MR* mitral regurgitation, *MV* mitral valve, *PHT* pulmonary hypertension.

*Other diagnosis: prior mitral valve repair or replacement.

### Postoperative outcomes

The median hospital stay was 14 days. Early mortality occurred in two patients (7%). (Table 2) The valve size and position did not affect the rate of postoperative heart block (P > 0.99, for both). Postoperative bleeding was not associated with the age group (P = 0.35), valve position (P = 0.32) or valve size (P = 0.71). Postoperative thrombosis or stroke were not associated with the age group (P > 0.99), valve position (P = 0.43), or valve size (P = 0.63).

**Table 2:** Postoperative outcomes.

Postop data	All patients (n = 29)	Age ≤ 24 months (n = 18)	Age > 24 months ≤ 60 (n = 11)	P
PHT	1 (3.5%)	1 (5.6%)	0	> 0.99
Postop inotropes	17 (58.7%)	11 (64.7%)	6 (54.6%)	0.70
Hemothorax	2 (6.9%)	1 (5.9%)	1 (9.1%)	> 0.99
Renal failure	1 (3.5%)	1 (5.9%)	0	> 0.99
Respiratory failure	1 (3.5%)	1 (5.9%)	0	> 0.99
Sepsis	6 (20.7%)	4 (23.5%)	2 (18.2%)	> 0.99
Heart block	7 (24.1%)	6 (35.3%)	1 (9.1%)	0.19
PPM	6 (20.7%)	5 (29.4%)	1 (9.1%)	0.36
**Thrombosis**				> 0.99
Valve	3 (10.3%)	2 (11.1%)	1 (9.1%)	
LV clot	1 (3.5%)	1 (5.6%)	0	
Stroke	2 (6.9%)	1 (5.6%)	1 (9.1%)	
ECMO	1 (3.5%)	1 (5.6%)	0	> 0.99
**Bleeding**				0.35
Subdural	3 (10.3%)	2 (11.1%)	1 (9.1%)	
Parenchymal	2 (6.9%)	2 (11.1%)	0	
Surgical	2 (6.9%)	0	2 (18.2%)	
Joints	1 (3.5%)	0	1 (9.1%)	
Minor	7 (24.1%)	5 (27.8%)	2 (18.2%)	
Hospital stay, days	14 (10–34)	14 (8–32)	16.5 (11.5–29.5)	0.79
Tracheostomy	0	0	0	
MV peak gradient	10 (6–14)	9 (6–12)	12 (9–19)	0.35
MV mean gradient	4 (3–7)	3 (2–6)	6 (4–9)	0.17
LVOTO	4 (13.8%)	1 (5.6%)	3 (27.3%)	0.14
**LV function**				0.58
Fair/good	19 (65.5%)	10 (55.6%)	9 (81.8%)	
Mild depressed	3 (10.3%)	2 (11.1%)	1 (9.1%)	
Moderately depress	4 (13.8%)	3 (16.7%)	1 (9.1%)	
Severely depressed	3 (10.3%)	3 (16.7%)	0	
Hospital mortality	2 (6.9%)	2 (11.1%)	0	0.51

*ECMO* extracorporeal membrane oxygenation, *LV* left ventricle, *LVOTO* left ventricular outflow tract obstruction, *PHT* pulmonary hypertension, *MV* mitral valve, *PPM* permanent pacemaker.

### Long-term outcomes

The median follow-up duration was 19.4 months (8.6–102.5). Survival was 93% at 1 year and 88% at 5 years. Among survivors, the 5-year freedom from reoperation was 81%. Nine patients had mitral valve reoperation (Five in Group 1 and four in Group 2). Six had MVR (three in each group), and three had clot removal from the mitral valve (two in Group 1 and one in Group 2). In addition, two patients underwent a second MVR. Mitral valve sizes used in the first reoperation were 25 mm (n = 1), 21 mm (n = 2), and 19 mm (n = 3). All patients had a larger-sized valve than their initial MVR except one patient with a dysfunctional mechanical valve and had the same size valve (19 mm), which was suitable for his body surface area. Patients with valve thrombosis had follow-ups in peripheral centers, and their INR records were not available.

**Figure 1:**
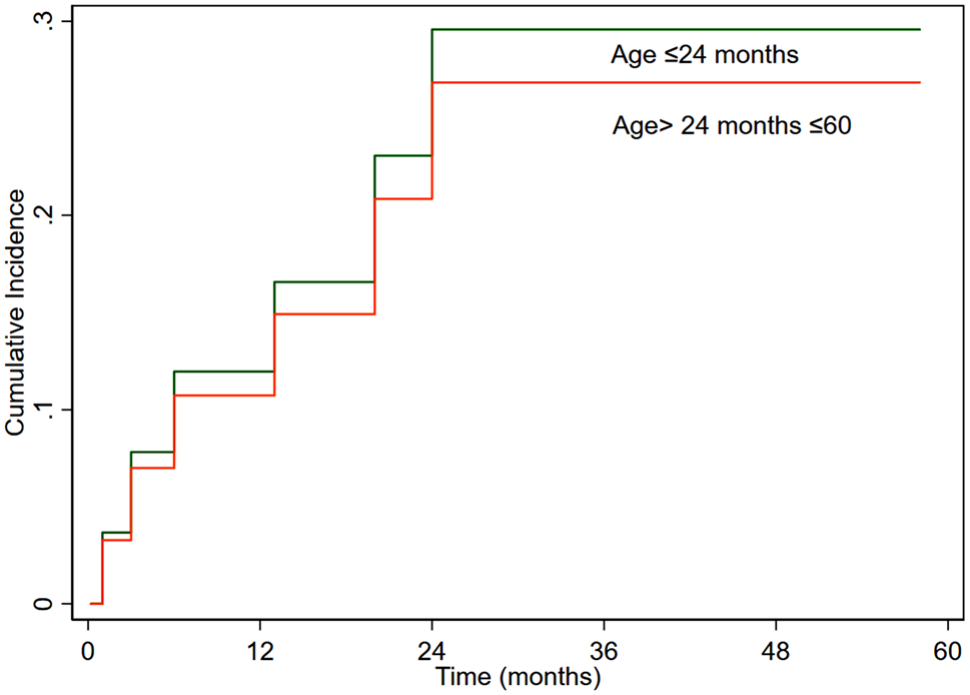
Cumulative incidence of mitral valve reoperation according to age.

**Figure 2:**
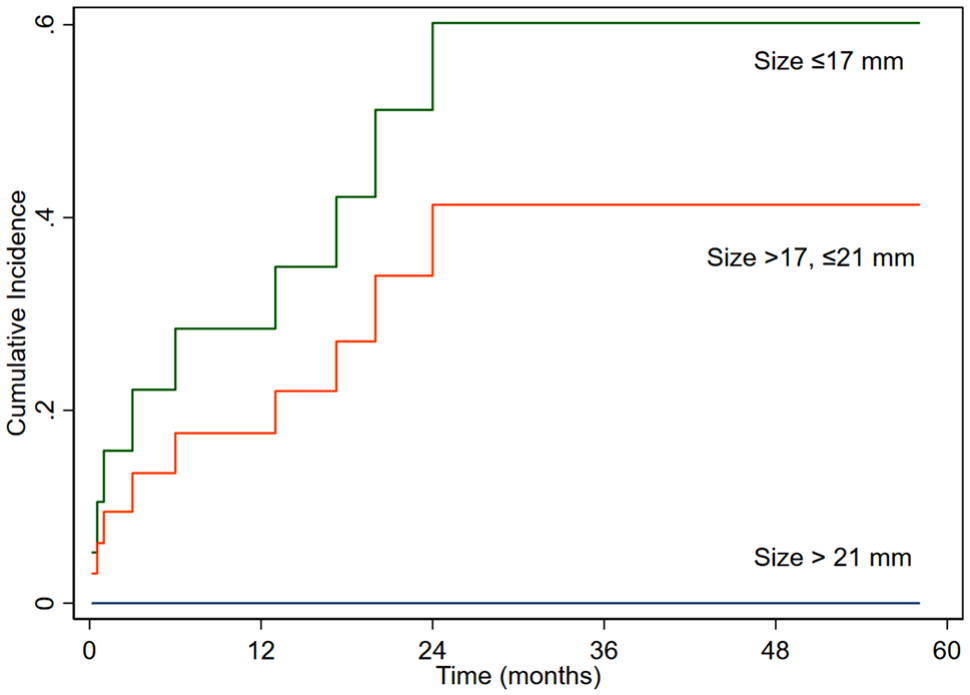
Cumulative incidence of mitral valve reoperation according to valve size.

**Figure 3:**
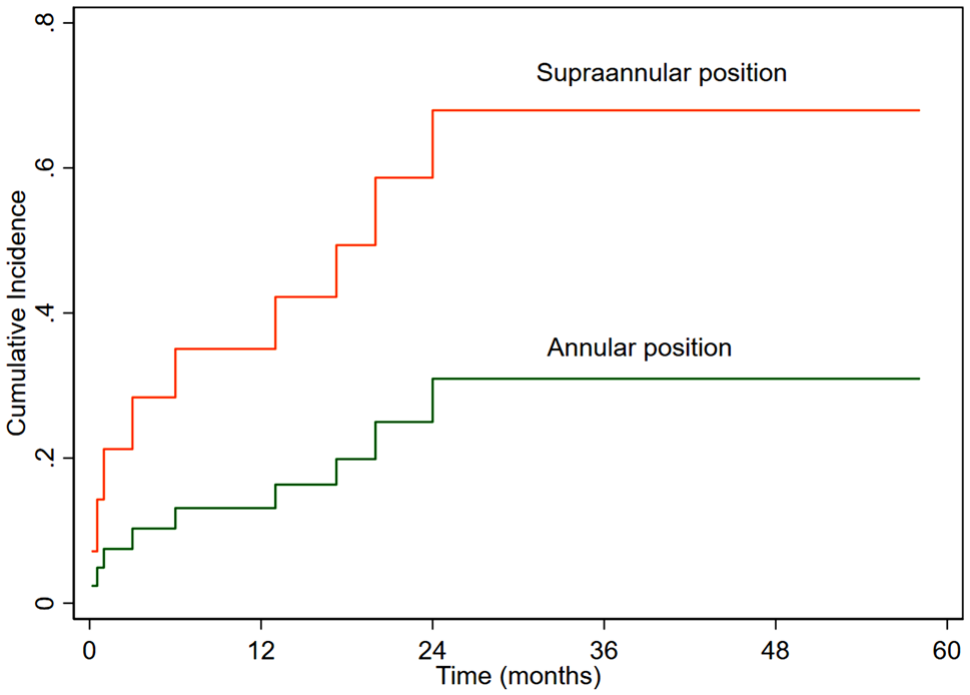
Cumulative incidence of mitral valve reoperation according to the valve position.

Competing risk analysis was performed to evaluate the effect of age on reoperation in the presence of death as a competing variable. We did not find effect of age group on reoperation (sub-hazard ratio (SHR) 0.89 (95% CI 0.27–2.87), P = 0.84) (Fig. 1). Valve size significantly affected reoperation (SHR 0.39 (95% CI 0.18–0.87), P = 0.02) (Fig. 2). Supra-annular position was associated with increased the risk of reoperation (SHR 3.1 (95% CI 1.003–9.4), P = 0.049) (Fig. 3). The preoperative diagnosis did not affect valve reoperation (SHR 1.14 (95% CI 0.52–2.46), P = 0.74).

**Figure 4:**
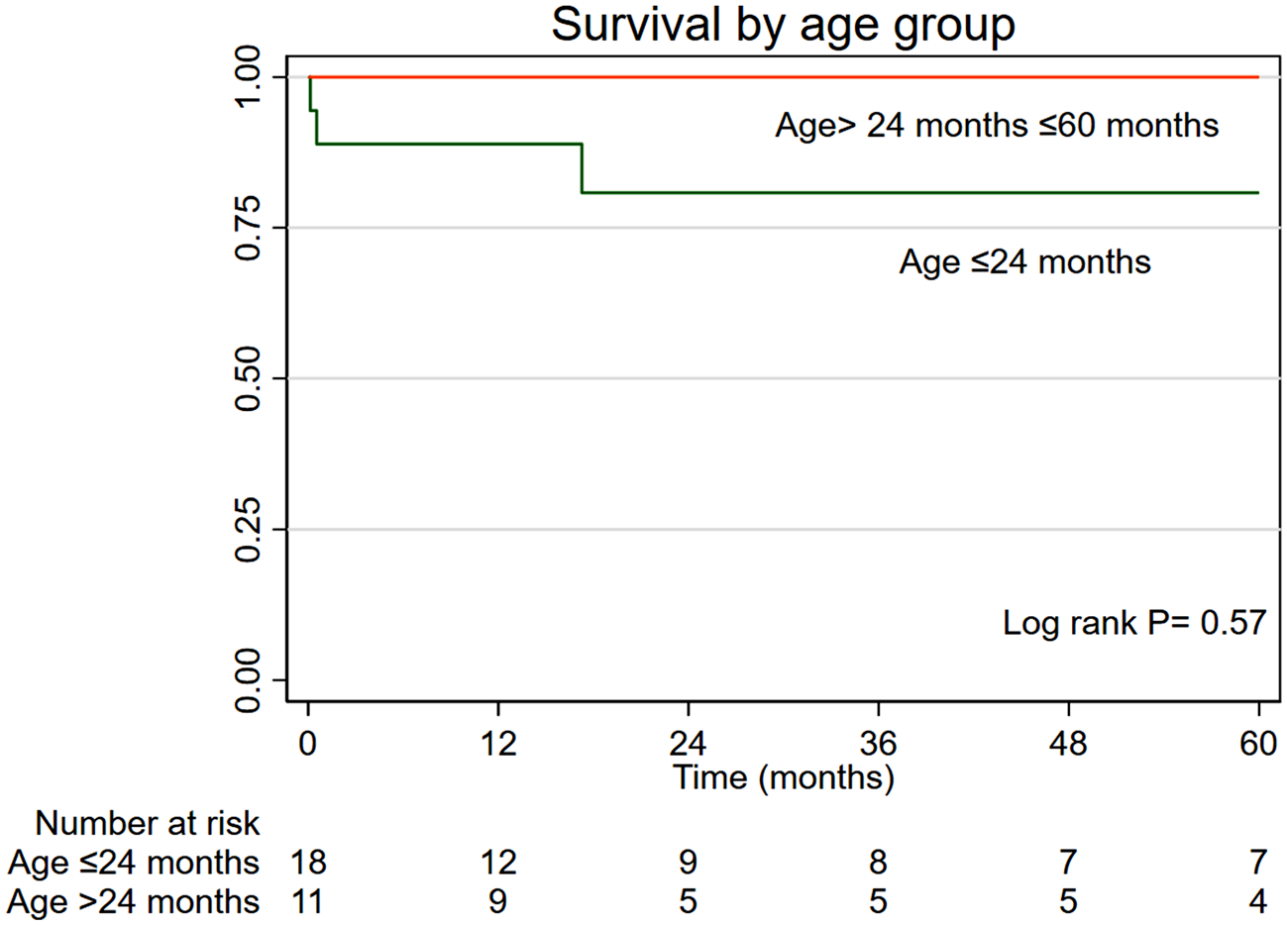
Kaplan–Meier survival curves according to age.

**Figure 5:**
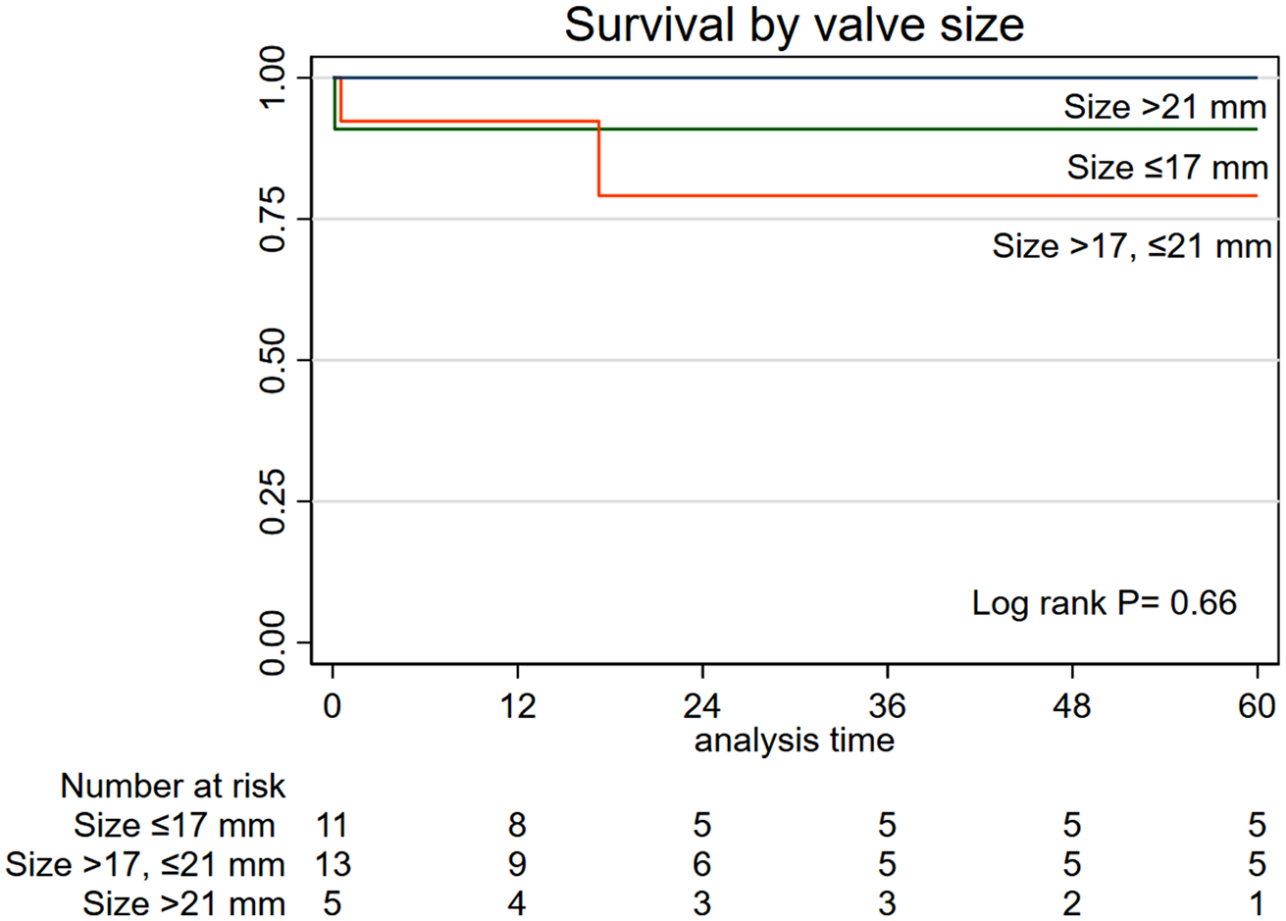
Kaplan–Meier survival curves according to valve size.

We did not find differences in survival according to the age (Log-rank P = 0.57) (Fig. 4), valve size (Log-rank P = 0.66) (Fig. 5), valve pathology (log-rank P = 0.39), diagnosis (Log-rank P = 0.49) and valve position (Log-rank P = 0.16).

## Discussion

Mitral valve replacement in infants and children is still a challenging procedure. The challenge arises from the unavailability of valve sizes, variable anatomy, the need for chronic anticoagulation, and child growth. Currently, valve repair is the standard procedure for surgical management of mitral valve diseases in children, and it is associated with low morbidity and better survival compared to MVR^6^. However, MVR remains a high-risk procedure with operative mortality rates of 0–36%^4,7,8^, despite the recent advances in surgical techniques and valves available. In addition, previous studies had shown increased mortality after MVR in children less than 5 years, and this mortality rate was even higher in children less than 2 years^9,10^.

The first challenge is the choice of adequate valve size for this critical age group. Supra-annular valve position is a surgical option to place a large-sized valve in children^11^. Another option suggested by Pluchinotta and colleagues is the stented bovine jugular vein graft (Melody valve) in the mitral position, which was found to be safe, effective, and reproducible, especially in small infants with the possibility of balloon dilatation later in life. However, at least in the short term, the morbidity and mortality risks are comparable to that of a mechanical prosthesis^12^.

Our study reported an overall mortality rate of MVR of 14%, which is similar to that reported by Henaine and associates with a similar number of patients and a mortality rate of 17%^13^. Caldarone and coworkers reported a mortality rate of 18% in the same age group^5^. However, our study's mortality rate was less compared to Selamet Tierney and associates, who reported a mortality rate of 39%^14^. The variability of mortality rates in the literature could be attributed to the included patients' age differences. In a study by Rafii and associates, age younger than 2 years was not associated with increased mortality risk after MVR^15^. Other factors that may have affected the survival are the valve size or position. In our study, the valve size or position did not affect the survival rate, which could be attributed to the small sample size. In another study, mortality was higher after MVR in children less than 2 years old^16^.

In our study, there was no correlation between valve size, age, or body surface area. This finding could be due to the unavailability of small valve sizes, and the adjustment of the valve size for different ages and weights was made by choice of valve position. We used the supra-annular position more frequently in infants less than 24 months old compared to the older age group. Supra-annular valve position was used in 20% of our study patients and was associated with an increased risk of reoperation but did not affect postoperative heart block. Selamet Tierney and coworkers used supra-annular position in 33% of cases with less risk of complete heart block but worse survival than the annular position^14^. Kanter and associates found that supra-annular position was not associated with postoperative heart block but had a high reoperation rate and pulmonary vein stenosis predicted the worse outcomes^17^.

The most common valve used in our study was St. Jude in 66% of cases. Selamet Tierney and colleagues reported the use of the St. Jude valve in 49% of cases and Carbomedics in 15% of cases^14^. Moreover, Caldarone and associates reported St Jude valve use in 79% of cases and Carbomedics in 7% of cases^5^. In our study, the size of MV used was less than 17 mm in 38%, 17–21 mm in 45%, and more than 21 mm in 17% of cases. The valve size in Selamet Tierney and coworkers' study was less than 17 mm 41%, 17–21 mm 32%, more than 21 mm 27% of cases^14^.

Indication for mechanical mitral valve replacement was 66% for MR, 10% for MS, and 24% for combined, similar to Caldarone et al., who reported MVR 65% for MR, 9% for MS, and 26% for combined^5^. The most common cause of MVR was congenital mitral valve disease, followed by Shone's complex. The etiological diagnosis may affect the outcomes after MVR^18^; however, we did not observe any effect in our study.

The postoperative heart block requiring pacemaker implantation in our study occurred in five patients (17%), which is comparable to Henaine and associates who reported heart block in four patients (14%)^13^. A similar rate was reported by Selamet Tierney and associates, where pacemaker implantation was required in 18% of patients^14^. Caldarone and coworkers in a similar age group reported the need for pacemaker insertion in 16% of cases^5^.

The non-significantly higher LVOT pressure in older age groups can be explained by the presence of more patients with Shone’s complex in this group, with undersized LVOT as part of their pathology.

Our study did not find a significant effect of age, weight, and valve type on the risk of reoperation; however, the smaller valve size was associated with a higher risk of reoperation. In the study by Henaine and associates, they reported a higher risk of reoperation with younger age, lower weight, Shone’s syndrome, smaller prosthesis, and prosthesis other than St. Jude^13^.

Our study is limited with the small sample size; however, mitral valve replacement in this age group is a rare procedure. In addition, the small sample size limited the statistical analysis, and several risk factors could have affected the outcomes and were not included in the analysis. Another limitation is the retrospective design with referral and selection biases. Moreover, the study is a single-center experience, and the generalization of the results needs further multi-center evaluation.

## Conclusion

Mitral valve replacement in children is associated with low morbidity and mortality, and the risk of reoperation could be affected by the valve size and position rather than the age.

## Data Availability

Patient data is available upon request with the corresponding author.
